# Disentangling Drivers of Food Waste in Households: Evidence from Nigeria

**DOI:** 10.3390/foods11081103

**Published:** 2022-04-12

**Authors:** Calvin Oluwafemi Sunday, Fatai Abiola Sowunmi, Oluwakemi Adeola Obayelu, Abiodun Emmanuel Awoyemi, Abiodun Olusola Omotayo, Adebayo Isaiah Ogunniyi

**Affiliations:** 1 Department of Agricultural Economics, University of Ibadan, Ibadan 200213, Nigeria; csfemi@gmail.com (C.O.S.); fa.sowunmi@ui.edu.ng (F.A.S.); oa.obayelu@ui.edu.ng (O.A.O.); 2 Department of Agricultural and Resource Economics, Faculty of Agribusiness and Applied Economics, University for Development Studies, Tamale P.O. Box TL 1350, Ghana; abiodunaw1996@uds.edu.gh; 3 Food Security and Safety Niche Area, Faculty of Natural and Agricultural Sciences, North West University, Private Bag X2046, Mmabatho 2790, South Africa; 4 International Fund for Agricultural Development (IFAD), Abuja 900101, Nigeria; a.ogunniyi@ifad.org

**Keywords:** food expenditure, food waste, rural household, beta regression model

## Abstract

Food waste is a burden on society in terms of the money wasted. There is limited information on the determinants of food waste and the amount lost to food waste by households as most previous studies were on post-harvest losses. Hence, determinants of food waste among households in Kogi West Senatorial District, Kogi State Nigeria were investigated. A three-stage sampling technique was used to select the respondents, while a structured questionnaire was used for data collection. Data were analyzed using Tobit regression and an equality test. The study revealed that food waste was higher in male headed households. The average monthly food waste proportion among urban households was significantly higher than that of rural households. The estimated amounts lost to food waste per month were ₦2103 and ₦5530 for the rural and urban households, respectively. These represented 7.2% and 13.1% of the total expenditure on food per month for rural and urban households, respectively. Among rural households, leftovers of food and lack of proper storage were the main reasons for food waste, while leftovers of food and preparation of food more than needed were the reasons for food waste among urban households. The sex of respondents, work experience, and monthly income influenced the proportion of food waste among rural households, while the dependency ratio, monthly income, and monthly food expenditure were the determinants of proportion of food waste among the urban households. Non-Governmental Organization efforts through sensitization campaigns focused on the need to reduce food waste, especially among urban households, would help to reduce the financial burden of food waste on households.

## 1. Introduction

Food is any substance that supplies energy for exercise, growth, and all physiological processes, as well as maintaining immune system health, and the amount of food a person requires varies from person to person depending on sex, age, and activity, among other factors [1,2]. Global Gastros [3] opined that humans destructively use food, and this is evident throughout history and present time; the wealthiest of societies would display their wealth through great banquets and feast, thus eating unhealthily, overeating, and wasting food while hunger was prevalent in society. Nigeria has a population of over 190 million people, and approximately 13 million Nigerians still suffer from hunger [4,5]. The global hunger index in Nigeria stands at 31.1 (serious), and the cereal import dependency ratio for Nigeria is 21.7 with a food deficit of 56 million tons [4,6].

According to [7], the solution to hunger is achieved through better use of the food that is already available, as much food produced for human use is lost or wasted. One-third of the food produced is wasted. Food waste is recognized as a distinct part of food loss because drivers that generate it are different from those that generate food loss. In developing countries, “food loss” or unintentional wastage is usually high due to poor equipment, transportation, and storage conditions [8]. Food loss emanates from the production, harvest, post-harvest, and processing phases while food waste is often caused by retailers and consumers throwing perfectly edible/spoilt foodstuffs into the trash [9]. However, this study centers around food waste.

Variation in the definitions of waste may result from what food waste consists of, how it is produced, and where and how it is being disposed. Furthermore, socio-cultural inclinations influence what is considered to be waste in some climates and otherwise in others, such as in the cases of visceral organs, leftovers, residues, peels, and others [10]. The Waste Resource and Action Programme [11] considers food waste to be a combination of the following: *Avoidable food waste, possibly avoidable food waste, and unavoidable food waste.* Food waste is the loss experienced at the retail and final consumption stage (consumer and households) of the food supply chain [12]. Per capita waste in Europe and North America is estimated to be between 95 and 180 kg per year, while in sub-Saharan Africa and Southeast Asia, it is between 6 and 11 kg per year. The United Nations in their response to reducing the rate at which food is wasted globally came up with Sustainable Development Goal 12, which is to “ensure sustainable and responsible consumption and production patterns”. This goal outlines a specific target for food waste reduction; that is, to halve per capita global food waste at the retail and consumer levels by 2030 [13]. Akerele et al. [14] reported that approximately 3–7% of food consumed per month, amounting to a monthly average value of ₦1500, is wasted by households in Nigeria, with root and tubers and cereals accounting for the largest percentage of total food waste.

Numerous studies in Nigeria [15,16,17] have focused their attention on postharvest losses and mitigation measures with little attention paid to studies on food waste among households. There is limited empirical evidence on the cost and causes of food waste in Nigeria. Given the prevailing scanty information on food waste problem in Nigeria, this study seeks to address this literature gap. The study is aimed at proffering solutions to the menace of food waste (reduction), thus removing pressure on scarce natural resources, decreasing the need to increase food production, and improving global food security, which are the focus of Sustainable Development Goal 2 [13]. Against this backdrop, the study investigates the determinants of food waste generation among households in Kogi West Senatorial District of Kogi State. The peculiarities of urban and rural households as they relate to food waste bring certain activities that contribute to food waste in our homes to the consciousness of households. The following research questions are raised, viz: What are the socioeconomic characteristics of the respondents in the study area? What is the sectoral proportion of food waste by respondents based on the educational status, household size, monthly income, and marital status? What is the average amount lost to food waste per month by sector in the study area? Is there a significant difference between the proportion of food waste by rural and urban households in the study area? What are the factors influencing the proportion of household food waste in the study area? For a detailed understanding of the study’s objective, the following research hypotheses were tested:

H_0_: *There is no significant variation in the average proportion of food waste between rural and urban households*.

H_0_: *The monthly income of respondents influences the proportion of food waste in the sector*.

## 2. Theoretical Framework and Literature Review

The theory of planned behavior has been widely applied to understanding consumer behavior and has shown to have the predictive power of attitudes, norms, perceived control, and intention on the behavior of the consumer. The theory of planned behavior developed by Ajzen [18] hypothesizes that “intentions to perform behaviour of different kinds can be predicted from attitude towards the behaviour, subjective norms and perceived behavioral control”. Attitudes represent an appraisal of self-performance of a behavior, subjective norms represent the perception of social pressure of how one should behave or not behave in a specific way, and perceived behavioral control represents the perceived ease of difficulty of behaving in a specific way [19,20]. Social norms and identity (traditions and culture), habits (eating habits, cooking skills, preservation skills among others), and external conditions (climatic and economic factors) can be employed to a large extent to explain the reasons behind food wastage in the household, such as in the case of a deliberate action from the consumer (behavior) or otherwise. Conceptually, using the theory of planned behavior as the framework for this study, food waste in households (behavior) can be linked to socioeconomic characteristics such as age, sex, income, household size, and marital status, among others (these make up their attitudes), as well as the number of meals eaten per day in the household, the frequency at which the household disposes of food, how the household eats food outside the home (habits), and the effect of external factors (perceived behavioral controls) such as climate, economic status, and the location of the household [21,22].

There are several analytical tools for measuring the determinants of food waste. The choice of method depends on the structure and scope of the study, the calculation techniques, and the units of measurement [23,24]. Food waste figures can be presented in several ways in relative terms to the total food production [25], total household expenditure, or budget on food [10,14]. Elawad et al. [26] used a logistic regression model to explore the association between perceived food waste generated within the household and the demographic variables while others [10] used probit regression to investigate the influence of socio-economic and demographic factors on household food waste. When a dichotomous variable serves as a dependent variable, there is relatively less variation in the predicted variable. Specifically, probit models require normal distributions for all unobserved components of utility. One of the shortcomings of probit and logit analyses is the relative lack of diagnostics that regression analysts have come to expect [27]. Using a dichotomous dependent variable may not be appropriate in this study since it is rare for a household to have 0% food waste (No food wasted). Stancu et al. [28] analyzed the determinants of consumer food waste behavior using Structural Equation Modeling (SEM). This model permits simultaneously modeling many relationships that use latent variables in the analysis as dependent or explanatory variables. However, the need for a large sample size for robust results is the limitation of the technique.

Visschers et al. [29] employed a Tobit regression model to analyze the total amount of self-reported food waste against several demographic variables. Moreover, Sun et al. [30] also analyzed the size and affect factors of household food waste in China. According to the Statistical Consulting Group [31], the Tobit model, also called a censored regression model, is designed to estimate linear relationships between variables when there is either left- or right-censoring in the dependent variable (also known as censoring from below and above, respectively). This makes Tobit regression unsuitable for fractional data such as the proportion of food waste by household. However, a type of fractional regression (beta regression, fractional probit model, fractional logit model, and fractional heteroskedastic probit model), which excludes zero and one in the dependent variable, called beta regression, was found to be appropriate for the study based of the available cross-sectional data. Beta regression, which addressed the rarity of a household not having food waste (0%) and having 100% food waste, was used. A major limitation of logistic regression is the assumption of linearity between the dependent and independent variables and thus can only be used to predict discrete functions. Generally, fractional regression does not require any special transformation of the values observed at the bounds and it is fully robust under generalized linear model assumptions [32].

### Analytical Framework of Beta Regression

The beta regression model was proposed by Ferrari and Cribrari-Neto [33] and Smithson and Verkuilen [34] for modeling covariate effects on a continuous response variable, which assumes support on the interval (0, 1). Beta regression is a type of fractional regression, which excludes 0 and 1 from its dataset [35,36]. Since it is impossible for a household to record 0% or 100% food waste over the period, beta regression is chosen. The beta regression model is a generalized linear model introduced by Ferrari and Cribari-Neto [33]. According to the authors, the probability beta density [*y* ~ B (*p*, *q*)] for the dependent variable *y* is defined in its general form as:

(1) $$f(y;p,q)=\frac{\Gamma \left(p+q\right)}{\Gamma \left(p\right)\Gamma \left(q\right)}y^{p-1}{\left(1-y\right)}^{q-1},\;0<y<1$$

where *p* and *q* are unknown parameters controlling the shape of the distribution, *p*, *q* > 0, *y* is a dependent variable, and Γ is the gamma function.

In beta regression, it is common practice to define the two shape parameters (*p, q*) of density to that of the mean (m) and precision parameter (w) [37]. After reparameterization to Equation (1) in terms of *µ* = *p*/(*p* + *q*) and ϕ=p+q, the probability beta density of a random variable *y* with a beta distribution $\left[y\sim B\left(\mu -\phi \right)\right]$ can be rewritten as [38]:

(2) $$f(y;\mu ,\phi )=\frac{\Gamma \left(\phi \right)}{\Gamma \left(\mu \phi \right)\Gamma \left(\left(1-\mu \right)\phi \right)}y^{\mu \phi -1}{\left(1-y\right)}^{\left(1-\mu \right)\phi -1},\;0<y<1.$$

where 0 < *µ* < 1 and *φ* > 0. From equation xx, the mean and the variance of the random variable y were defined as E(y)=μ and $Var(y)=\mu \left(1-\mu \right)/\left(1+\phi \right)$. For the precision parameter $\left(\phi \right)$ of a fixed estimate (mean), the higher the ϕ value, the smaller the variance of the variable [39]. Assuming the percentage response variables were beta-distributed, a beta regression model is designed. Let *y*_1_, *y*_2_, …, *y*_n_ be a random sample from beta density $B\left(\mu_{i},\phi \right)\;\left[y\sim B\left(\mu_{i},\phi \right)\right]$. Cepeda-Cuervo [39] defined beta regression as:

(3) $$g\left(\mu_{i}\right)=\beta_{0}+x_{i1}\beta_{1}+\ldots \ldots \ldots \ldots \ldots \ldots +x_{ik}\beta_{k}=\eta_{i},\;i=1,\ldots ,\;n$$

where *x_i_*_1_, …, *x_ik_* are the covariates, *b*_0_, *b*_1_, …, *b_k_* are the estimated intercept and coefficients corresponding to each covariate, *η_i_* is the linear predictor for the *ith* observation, and *n* is the sample size.

Here, $g\left(.\right)$ is a link function, which connects the linear predictor and the response variable. The logit link was used in this study $\left[g\left(\mu \right)=\log\left(\mu /\left[1-\mu \right]\right)\right]$ for beta regression.

## 3. Methodology

### 3.1. Description of the Study Area

**Figure 1:**
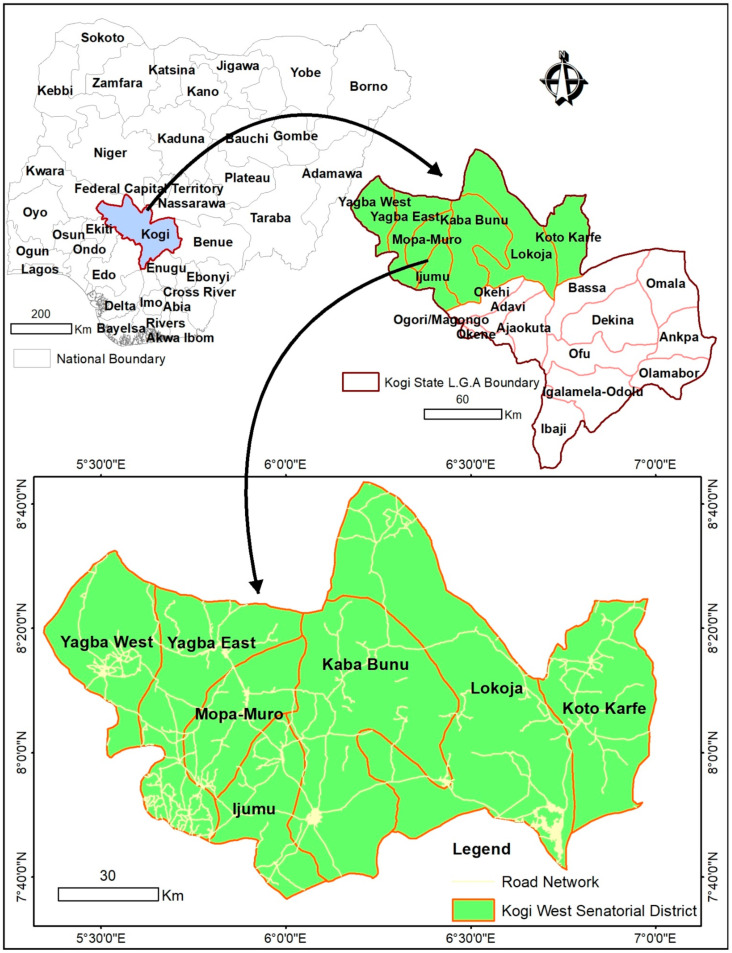
Geographical location of the selected communities in Kogi West Senatorial District of Kogi State, Nigeria. Source: Authors.

The study was conducted in Kogi West Senatorial District (SD) of Kogi State located in the North Central Geo-political zone of Nigeria in 2021. It is located between longitudes 5°0′21″ E–7°0′0″ E and latitudes 7°00′30″ N–8°0′50″ N (Figure 1). Kogi West Senatorial District is made up of seven Local Government Areas (LGAs), namely Yagba West, Yagba East, Mopa Moro, Ijumu, Kabba/Bunu, Lokoja, and Kogi LGAs (Kogi State Government, 2019). Lokoja and Kabba/ Bunu LGAs are classified as urban in terms of socio-economic factors, infrastructures, and development with a large presence of traders, private businesses, and civil servants while others (Yagba West, Yagba East, Mopa Moro, Ijumu, and Kogi LGAs) are relatively rural [40]. On the whole, Kogi west senatorial district is heterogeneous in nature with diverse cultures and languages among which are the Okun (major ethnic group), Bassa Nge, Egbura Koto, Gwari, and Kakanda, among others [41]. Kogi West Senatorial District is estimated to have a total population of 906,244 persons with Lokoja and Mopa Moro LGAs being the largest and smallest LGAs in the senatorial district, respectively, and it occupies 12,498.422 km^2^ of arable land (National Population Commission, 2010; NBS, 2010). Its climate is characterized by wet and dry seasons. Most residents of rural areas of this senatorial district area are farmers. The SD is known for the production of crops such as yam, cassava, soya bean, cocoyam, maize, millet rice, guinea corn, and oil palm cashew [42,43].

### 3.2. Sample Selection and Data Collection

A three-stage sampling technique was employed. The first stage involved randomly selecting two LGAs within the study area; these were Lokoja and Yagba East LGAs. The second stage involved a purposive selection of towns and villages within the selected LGAs proportionate to their population sizes. The purposive selection of towns and villages was performed to make provisions for categorizing respondents into urban and rural areas. Thus, 17 towns and villages were selected in Yagba East LGA while 21 towns and villages were selected in Lokoja LGA. For Yagba East LGA, three urban areas and fourteen rural areas were selected while five urban areas and sixteen rural areas were selected for Lokoja LGA. Furthermore, respondents (households) were randomly selected proportionate to the size of their towns and villages across the rural and urban areas to give a total of 270 respondents (households). Primary and secondary data were used in the study. Primary data were collected using structured questionnaires. The data collected included socioeconomic characteristics (age, sex, marital status, household size, number of people working in the household, educational status, monthly income, and occupation of household head), number of meals per day, number of times eating out per week, monthly household food expenditure, percentage of monthly food expenditure wasted, reasons for food waste in the household, common food item wasted, method of disposing of food not consumed, number of times of disposing of unconsumed food per week, season of the year when food spoilage is high, and if more food is wasted during festive periods. Out of the 270 copies of questionnaire administered, 267 were suitable for analysis, while 3 questionnaires were not properly completed. However, secondary data were elicited from journals and online publications.

The calculated sample sizes (respondents: 245.9 ≅ 246) for the study were obtained using International Fund for Agricultural Development (IFAD) procedure based on the formula below. The final sample size (270) used included allowances for the design effect and contingency. The allowance for design effect is expected to correct for the difference in design while the allowance for contingency accounts for contingencies such as non-response or recording error. The sample sizes were obtained using:

(4) $$n=\frac{z^{2}p(1-p)}{m^{2}}$$

where *n* is the sample size, *Z* is the confidence level at 95% (1.96), and *p* is the estimated %age of respondents willing to partake in the study

#### Data Analysis

Data were analyzed using descriptive statistics to profile the socioeconomic characteristics of the respondents and describe the food waste situation in the study area. The descriptive analyses employed were a measure of central tendency (mean), a measure of dispersion (standard deviation and skewness), and the frequency distribution. In this study, the respondents doubled as the household heads. The equality test was used to examine if there was significant difference in food waste proportion between rural and urban households. This is achieved given that:

(5) $$Z=\frac{{\overset{⌢}{p}}_{rur}-{\overset{⌢}{p}}_{urb}}{\sqrt{\overset{⌢}{p}\left(1-\overset{⌢}{p}\right)\left(\frac{1}{n_{rur}}+\frac{1}{n_{urb}}\right)}}\;$$

where ${\overset{⌢}{p}}_{rur}$ is the average food waste proportion among urban households, ${\overset{⌢}{p}}_{urb}$ is the average food waste proportion among urban households, $\overset{⌢}{p}$ is the average proportion of food waste among rural and urban households (pooled proportion), $n_{rur}$ is the number of rural households, and $n_{urb}$ is the number of urban households.

Beta regression was used to determine the factors influencing food waste proportions among households (rural and urban) in the study area. The model was specified as follows:

(6) $$Y=\beta_{0}+\beta_{1}X_{1}+\beta_{2}X_{2}+\beta_{3}X_{3}+\beta_{4}X_{4}+\beta_{5}X_{5}+\beta_{6}X_{6}+\beta_{7}X_{7}+\;\beta_{8}X_{8}+\beta_{9}X_{9}+\beta_{10}{\;X}_{10}+\beta_{11}X_{11}+\beta_{12}{\;X}_{12}+\beta_{13}X_{13}+\beta_{14}X_{14}+\mu_{i}$$

where *Y* is households’ proportion of food waste per month per household; *X*_1_ is the sex of the respondent (0 = Female, 1 = Male); *X*_2_ is the age of the respondent (years); *X*_3_ is the marital status of the respondent (0 = Not married, 1 = Married); *X*_4_ is respondents’ years of schooling (years); *X*_5_ is working experience (years); *X*_6_ is household size; *X*_7_ is the dependency ratio $\left(Dependency\;ratio\;=\;\frac{Number\;of\;dependants}{Number\;of\;households\;members\;working}\;x\;\frac{100}{1}\%\right)\;$; *X*_8_ is the monthly income of household head (₦); *X*_9_ is households’ monthly food expenditure (₦); *X*_10_ is the average number of meals per day; *X*_11_ is the number of times household eat out per week; *X*_12_ is the number of times food not consumed is disposed of per week by the household; *X*_13_ is food waste during seasons of the year (0 = Dry season, 1 = Raining season); *X*_14_ is the food waste during festive periods (1 = Yes, No = 0); and *U*_i_ is the error term.

## 4. Results and Discussion

### 4.1. Socioeconomic Characteristics of Respondents

Table 1 shows that 46.3% and 67.5% of respondents/household heads in rural and urban areas, respectively, were male, while female respondents/household heads were 53.7% and 32.5% in rural and urban areas, respectively. The table revealed that male-headed households (9.07%) generally had a greater tendency to waste food (9.93%) compared to female-headed households. Secondi et al. [44] and Cantaragiu [45] affirmed that women tend to be more conscious of the negative impact of food waste on household expenditure, thus making them less likely to generate food waste compared to men. The age distribution shows that 27.9% and 25.8% of the rural and urban respondents were within the age bracket of 38–47 years. The average respondent ages were 43.2 and 42.5 years for rural and urban respondents, respectively. The average ages imply that most respondents in the study area were in their productive ages, which is in accordance with [46,47,48]. Moreover, respondents in the age bracket of 27–37 years had the highest proportion of food waste per month (10.44%).

The majority of the respondents in rural (65.3%) and urban (66.7%) households were married, while 21.1% and 25.8% were in single households in rural and urban areas, respectively. Married households wasted less food (9.52%) compared to single households (10.56%). A high literacy level was observed among the respondents, as 60% of the respondents in urban areas were B.Sc./HND certificate holders while 37.4% of respondents in rural areas had NCE/OND certificates. Moreover, 3.4% of the respondents in rural areas had no formal education. Respondents who had only primary education wasted less food in both rural and urban households. This may be attributed to the paucity of resources at their disposal. The household size ranged from 1–20 persons. Approximately 61% and 58.3% of rural and urban households, respectively, had between 1 and 5 persons per household, while the average household sizes were 5 persons and 5.2 persons, respectively, for rural and urban respondents. The study also showed that 76.2% of rural respondents and 88.3% of urban respondents had at least three dependents per household. Contrary to expectations in poor household with a large family size, the result showed that the larger household size had a higher proportion of food waste per month.

Furthermore, Table 1 shows that 50% of the respondents in urban areas were civil servants while 16.7% engaged in other economic activities. The number of respondents that engaged in farming (19.7%) as their main occupation was lower than that of civil servants (32.7%) and traders (22.4%) in the rural areas. Moreover, the average monthly incomes for rural and urban respondents were ₦40,816.43 ($98.40) and ₦99,375.36 ($239.58), respectively. Among rural households, 78.9% earned, at most, ₦50,000 per month, while 59.2% of urban households earned between ₦50,001 and ₦150,000 per month. The average household monthly food expenditures were ₦29,133.14 ($70.24) and ₦42,250.49 ($101.86) for rural and urban households, respectively. The proportion of food waste was highest among respondents with monthly food expenditure of at least ₦105,001. The study revealed that the respondents with monthly income more than ₦50,000 ($120.54) had food waste of at least 11.72% per month in the study area. In addition, 71.4% and 42.5% of the respondents’ monthly income were spent on food in rural and urban areas, respectively (average monthly food expenditure (rural/urban) was divided by the average monthly income (rural/urban)). This finding agrees with previous research [7,17] and Engel’s law that poor households (more common in rural areas in Nigeria) spend a larger share of the household income on food.

### 4.2. Critical Factors for Food Waste Generation

Table 2 shows that 53.1% and 63.3% of rural and urban respondents, respectively, ate between 3 and 4 meals per day. Households that consume more meals per day had a higher proportion of food waste as it brings about more leftovers. The study showed that 53.3% of urban households ate food outside the house (canteen, eatery) at least once a week. On the contrary, only 23.3% of rural households ate food outside home. Moreover, Table 2 shows that 61.2% and 57.5% of the respondents in rural and urban areas, respectively, claimed their proportion of food waste was light. Among the rural area respondents, 67.3% attributed food waste to leftovers of food while 53.7% adduced the lack of proper storage to causing food waste. Conversely, for urban households, 52.5% and 46.7% attributed food waste to leftovers of food and the preparation of more food than needed, respectively. Other reasons given by respondents for food waste were the burning of food, expiration date, and wrong preservation method.

The study revealed that 54.4% of rural households fed leftovers and food not consumed to pets and domestic animals, while 22.0% of the urban households gave out food not consumed to people around them, especially to domestic staff and the less privileged (see Table 2). The study affirmed that 70.1% and 63.3% of the rural and urban respondents claimed that food waste was more pronounced during the rainy season, while 74.8% and 60.0% of the respondents in the rural and urban areas, respectively, believed that a large quantity of food was wasted during the festive period. The pronounced food wastage during the rainy season may be attributed to high atmospheric moisture, which provides favorable conditions for microbial growth and insect infestation on food items. For example, fungi multiply greatly in damp and warm condition on cereals and grains in storage and thus spoil easily if not properly preserved [49].

**Figure 2:**
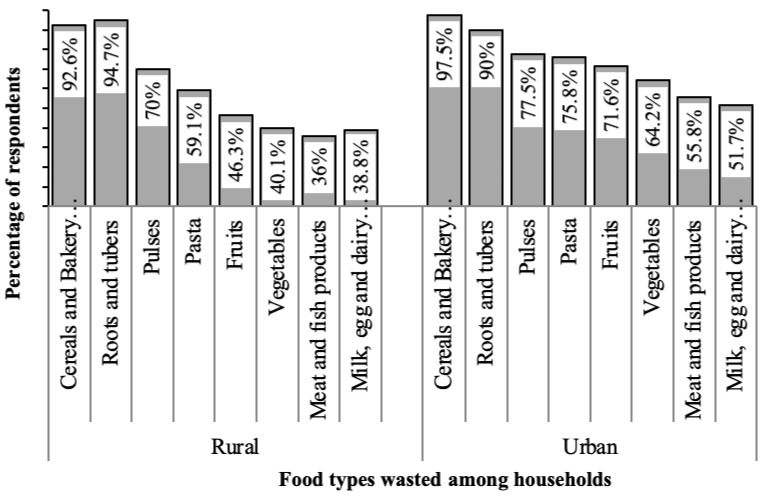
Distribution of common food types wasted among households in the study area.

The most common food wasted among rural households was root and tubers, while cereals and bakery products were frequently wasted by urban households (see Figure 2). Roots and tubers constitute 94.7% of the food waste in rural area, while cereals and bakery products amounted for 97.5% of food wasted in urban areas. This agrees with the findings of Horodyska et al. [50] that rural areas reported significantly higher mean daily intakes of tubers (122 g/day) among tuber consumers than urban areas (95 g/day). Roots and tubers have long been a source of food and nutrition and, thus, common among poor and malnourished households. Roots and tubers are generally valued for their stable yield in conditions under which other crops may not thrive [51,52,53], and are a basic source of low-cost energy food. For example, cassava is a cheap source of carbohydrates and a valuable source of cheap calories, especially among poor households [54]. Furthermore, urban households, over time, revealed a preference for more convenient foods, and this can be attributed to the availability and ease of importing of low-cost cereals coupled with higher purchasing power.

Furthermore, Table 3 shows that the proportion of food waste per month in the study area was 9.5%. The average food waste proportion per month among households in rural and urban areas was 7.1% and 12.5%, respectively. This amounted to ₦2103 ($5.07) and ₦5530 ($13.32) per month for rural and urban households, respectively. The result affirmed that 7.2% and 13.1% of the monthly expenditure on food was lost to food waste in rural and urban households, respectively. This is in agreement with Taghipour et al. [55], who suggested that urban households waste more food than rural households because of the higher wealth profile and the need to have food stored at home for some time after purchasing, unlike rural households that harvest crops for immediate needs.

### 4.3. Variation in the Average Food Waste Proportion between Urban and Rural Households

Table 4 shows that there was significant variation between the average proportion of food waste between urban and rural households (*p* < 0.01). That is, the average food waste proportion in the urban households was significantly greater than the average food waste proportion in rural households. For example, some urban housing settings (housing estates) had no market facility within, which, at times, made respondents buy food items in bulk and more than what was needed at the time for convenience/time-saving reasons, and thus often increases the proportion of food waste. Furthermore, the patronage of fast-food services by urban households was identified as a factor that has led to an increase in the proportion of food waste among households. Moreover, some rural dwellers in the study area revealed that, due to the low income and poor finance experienced by the households, the proportion of food wasted was less. According to Secondi, Principato, and Laureti [44] and Jiang et al. [20], differences in household food waste along the divide of te rural–urban continuum can also be traced to the different culture, environmental differences, wealth profile, cooking styles and techniques, dietary structure, and consumption habits prevalent in the regions and respective households. This corroborates the findings of Chakona and Shackleton [54] and Taghipour et al. [55] that household food waste in urban settings is higher than that in rural settings. Furthermore, the disaggregation of the proportion of food waste among some selected socioeconomic variables further emphasizes the variation in food waste proportion between rural and urban households (see Table 2).

### 4.4. Determinants of Food Waste Proportion among Households in the Study Area

The determinants of the proportion of food waste among households in the study area were disaggregated into the rural and urban households (respondents) and the results presented accordingly in Section 4.4.1 and Section 4.4.2.

#### 4.4.1. Determinants of Food Waste Proportion among Rural Households

Table 5 shows the beta regression results for the rural and urban households in the study area. The LR ch^2^ was significant (*p* < 0.01). This affirms that the model has good fit. model. The marginal effects of the sex of the respondent, work experience, household dependency ratio, monthly income of household head, and the number of times a household disposes of food not consumed per week were significant at various levels. Specifically, the result shows that for every male head of household, the proportion of food waste increased by 0.013 (*p* < 0.05). This implies that by being a male-headed household, the proportion of food waste increases by 1.3%. This affirms the finding of Akerele, Afolayan, Oyawole, and Sanusi [14] that male-headed households wasted more fruits and vegetables in their households compared to female-headed households. Furthermore, respondents’ work experience (years) had a negative relationship with the proportion of household food waste (*p* < 0.01). A unit increase in the years of work experience of the respondent will decrease household food waste by 0.17%.

The study also showed that the dependency ratio reduces the proportion of food waste by 0.36%. That is, as the dependency ratio increases, the proportion of food waste decreases. This result agrees with Qi and Roe [56] that a positive relationship exists between the number of household members employed/engaging in economic activities and the food waste level in the household. Moreover, the monthly income of the respondent revealed a positive relationship with the proportion of food waste (*p* < 0.05). This indicates that for every naira increase in the monthly income of respondent, the proportion of food waste increased marginally (0.000023%). This finding is affirmed by Stancu, Haugaard, and Lähteenmäki [28] and Sun [30] that income and household food waste exhibited positive relationships. The Waste Resource and Action Programme [11] posited that low-income households wasted more food because they do not plan for their shopping and have a “live for today” attitude. However, Koivupuro et al. [57] and Visschers, Wickli, and Siegrist [29] reported no relationship between income and food waste. Furthermore, the number of times food not consumed was disposed of per week in the household had a positive relationship with the proportion of food waste (*p* < 0.05). Schane, et al. [58] opined that respondents who discard food occasionally produce less food waste in their household in contrast to those who throw away food every time.

#### 4.4.2. Determinants of Food Waste Proportion among Urban Households

As shown in Table 5, the diagnostic result for urban households shows that the LR Ch^2^ test was significant. This means that the model has good fit. Among the urban households, the marginal effects of the dependency ratio, monthly income, household’s expenditure on food, household’s average number of meals per day, number of times a household eats out, and the number of time household dispose of food not consumed were significant.

The household dependency ratio had a negative relationship with the households’ food waste proportion. This indicates that a unit increase in te household dependency ratio will reduce the food waste proportion in the household by 1.7%. As observed by Qi and Roe [56], the number of household members employed/engaging in economic activities and food waste level in the household are positively related. The monthly income of the household head, the household food expenditure per month, the number of times the household eats per day, the number of time the household eats away from home, and the number of time food not consumed is thrown away had positive relationship with the proportion of food waste per week by household. The positive relationship between the household head’s income per month and the proportion of food waste is in agreeance with Stancu et al. [28] who suggest households with higher income tend to buy more food and the tendency to waste food increases compared to low-income households. Moreover, Parizeau et al. [59] and Abdelradi [60] affirmed that households that spend more money on food items would produce more food waste. Parizeau et al. [59] observed that cooking and eating at home reduces the reliance on ordering food or fast foods and eating out, and thus would result in reduced food waste in households. Mallinson et al. [61] suggested that those consumers who rely heavily on convenience food, including ready-made, fast meals, and restaurant takeaways, tend to waste more food than others.

## 5. The Study Limitations

The study relied solely on the information from 270 selected households in the Kogi West Senatorial District of Kogi State, Nigeria and not the entire population of Kogi State. More so, out of all the rural and urban communities in the state, some were randomly selected that cannot be taken as a general representation of the determinants of food waste among households in Kogi State or Nigeria as a whole. Furthermore, the current study focused on households in the selected communities and not the entire households. Finally, the effect of food waste on the environment was not included in the study and this is a limitation of the study. 

## 6. Conclusions and Recommendations

There was substantial food waste among the households in the study area. The money lost to food waste monthly confirmed that food waste was an economic burden on households. Eradication of food waste may not be achievable, but its reduction will not only relieve the burden on households but lessen the pressure on natural resources used in food production. The problem associated with the use of refrigerators was common in urban areas due to poor public power supply. Generally, households in the study area often fail to plan their food purchases/needs properly, which results in food waste. The food waste was higher among urban households. Non-governmental organizations’ efforts through sensitization campaigns focused on the need to reduce food waste, especially among urban households, would help to lessen the economic burden of food waste. The need for affordable food preservation techniques should be part of feedback extension agents discussed during monthly technical review meetings to reduce food waste in the study area.

## Figures and Tables

**Table 1:** Socioeconomic characteristics and the proportion of food waste per month by respondents.

Socioeconomic Characteristics	Rural			Urban			Total		
	Freq.	%	Proportion of FW	Freq.	%	Proportion of FW	Freq.	%	Proportion of FW
Sex of respondents									
Male	68	46.3	7.53	81	67.5	11.95	149	55.8	9.93
Female	79	53.7	6.77	39	32.5	13.73	118	44.2	9.07
Marital status									
Single	31	21.1	7.80	31	25.8	13.39	62	23.2	10.56
Married	96	65.3	7.16	80	66.7	12.36	176	65.9	9.52
Divorced	3	2.0	7.33	4	3.3	15.00	7	2.6	11.71
Widowed	17	11.6	5.65	5	4.2	7.80	22	8.2	6.14
Level of education									
No formal education	5	3.4	6.40	-	-	-	5	1.9	6.40
Primary school	16	10.9	5.88	3	2.5	8.33	19	7.1	6.26
Secondary education	40	27.2	7.88	4	3.3	9.50	44	16.5	8.02
OND/NCE	55	37.4	6.73	22	18.3	12.11	77	28.8	8.27
HND/BSc	29	19.7	7.23	72	60.0	12.90	101	37.8	11.28
Postgraduate	1	0.7	15.00	19	15.8	12.90	20	7.5	13.00
Others	1	0.7	10.00	-	-	-	1	0.4	10.00
Main occupation									
Civil Servant	48	32.7	7.04	60	50.0	12.23	108	10.4	9.92
Artisan	23	15.6	7.57	16	13.3	13.91	39	14.6	10.17
Farming	29	19.7	7.10	-	-	-	29	10.9	7.10
Private business	13	8.8	8.46	20	16.7	12.30	33	12.4	10.79
Trading	33	22.4	6.45	20	16.7	12.20	53	19.9	8.62
Retired	1	0.7	6.00	4	3.3	14.25	5	1.9	12.60
Monthly income									
At most 50,000	116	78.9	6.82	33	27.5	9.98	149	55.8	7.52
50,001–150,000	31	21.1	8.23	71	59.2	13.23	102	38.2	11.72
150,001–250,000	-	-	-	12	10.0	13.68	12	4.5	13.68
250,001–350,000	-	-	-	1	0.8	25.00	1	0.4	25.00
350,001–450,00	-	-	-	2	1.7	12.50	2	0.7	12.50
450,001–550,000	-	-	-	1	0.8	20.00	1	0.4	20.00
Age									
18–27	14	9.5	7.00	13	10.8	11.27	22	10.1	9.06
28–37	37	25.2	7.68	33	27.5	13.54	70	26.2	10.44
38–47	41	27.9	7.15	31	25.8	12.45	72	27.0	9.43
48–57	36	24.5	7.25	29	24.2	11.79	65	24.3	9.28
58–67	17	11.6	5.82	12	10.0	14.38	29	10.9	9.36
68 and above	2	1.4	6.00	2	1.7	4.50	4	1.5	5.25
HH size									
1–5	90	61.2	7.05	70	58.3	13.17	160	59.9	9.72
6–10	56	38.1	7.30	47	39.2	11.41	103	38.6	9.17
11–15	1	0.7	5.00	2	1.7	15.00	3	1.1	11.67
16 and above	-	-	-	1	0.8	15.00	1	0.4	15.00
Dependency ratio									
0–3	112	76.2	7.50	106	88.3	12.73	218	81.6	10.04
4–6	29	19.7	6.07	10	8.3	12.90	39	14.6	7.82
Above 6	6	4.1	5.33	4	3.3	6.13	10	3.7	5.65
HH food expenditure (₦)									
At most 15,000	4	2.7	7.75	2	1.7	6.25	6	2.2	7.25
15,001–30,000	84	57.1	7.00	31	25.8	10.70	115	43.1	8.00
30,001–45,000	50	34.0	6.74	48	40.0	12.74	98	36.7	9.68
45,001–60,000	8	5.4	9.50	26	21.7	13.15	34	12.7	12.29
60,001–75,000	1	0.7	15.00	-	-	-	1	0.4	15.00
75,001–90,000	-	-	-	6	5.0	16.33	6	2.2	16.33
90,001–105,000	-	-	-	6	5.0	12.92	6	2.2	12.92
105,001 and above	-	-	-	1	0.8	30.00	1	0.4	30.00
No. of children between 2–10 years									
0–2	121	82.3	7.08	100	83.3	12.59	221	82.8	9.58
3–5	24	16.3	7.29	17	14.2	11.99	41	15.4	9.24
6 and above	2	1.4	7.50	3	2.5	13.33	5	1.9	11.00
Total	147	100	7.12	120	100	12.52	267	100	9.45

NB: FW means food waste.

**Table 2:** Critical factors in food waste generation.

Characteristics	Rural			Urban			Total		
	Freq.	%	Proportion of FW	Freq.	%	Proportion of FW	Freq.	%	Proportion of FW
Average number of meals per day among households									
1–2	67	45.6	7.14	41	34.2	11.07	108	40.4	8.63
3–4	78	53.1	7.09	76	63.3	13.15	154	57.7	10.08
5 and above	2	1.4	8.00	3	2.5	16.67	5	1.9	13.2
Average number of times eating out per week									
Never	75	51.0	6.56	41	34.2	10.81	116	43.4	8.06
Less than 2 times	56	38.1	7.66	64	53.3	12.75	120	44.9	10.37
2–4 times	14	9.5	7.93	13	10.8	16.31	27	10.1	11.96
Above 5 times	2	1.4	7.50	2	1.7	16.00	4	1.5	11.75
Household food waste self-categorization									
Light	90	61.2	6.38	69	57.5	11.31	159	59.6	8.52
Moderate	47	32.0	8.43	46	38.3	13.91	93	34.8	11.14
Heavy	10	6.8	7.70	5	4.2	16.50	15	5.6	10.63
Methods of disposing food not eaten by household									
Feed it to pets and animals	59	47.6	7.24	42	37.2	11.39	101	42.6	8.97
Give it out	28	22.6	6.46	26	23.0	11.09	54	22.8	8.69
Throw away/dispose	35	28.2	6.80	41	36.3	14.20	76	32.1	10.79
Others	2	1.6	8.00	4	3.5	12.50	6	2.5	11.00
Average number of times food is disposed per week									
At most 2	116	78.9	6.59	74	61.7	10.86	190	71.2	8.25
3–4	29	19.7	9.21	32	26.7	15.58	61	22.8	12.55
5 and above	2	1.4	8.00	14	11.7	14.36	16	6.0	13.56
Food spoilage and season of the year									
Raining	103	70.1	7.17	76	63.3	12.41	179	67.0	9.36
Dry	36	24.5	7.11	38	31.7	12.57	74	27.7	9.92
Harmattan	8	5.4	7.38	6	5.0	13.67	14	5.2	10.07
Food waste and festive period									
Yes	110	74.8	7.35	72	60.0	12.91	182	68.2	9.55
No	33	22.4	6.43	39	32.5	11.65	72	27	9.26
I do not know	4	2.7	6.50	9	7.5	13.24	13	4.9	11.18
Reasons for food waste among households									
Leftover foods	99	67.3		63	52.5		162	59.9	
Lack of proper storage	79	53.7		39	32.5		118	43.1	
Preparing more than the need	53	36.1		56	46.7		109	41.4	
Burning of food	33	22.4		20	16.7		53	19.6	
Buying too much	17	11.6		24	20.0		41	15.8	
Bad quality	14	9.5		19	15.8		33	12.7	
Wrong preservation method	28	19.0		2	1.7		30	10.4	
Growth of mold	4	2.7		4	3.3		8	3.0	
Expired food	1	0.7		4	3.3		5	2.0	

NB: FW means food waste.

**Table 3:** Distribution of household food waste proportion per month.

Proportion (%) of Household Food Waste/Month	Rural		Urban		Total	
	Frequency	%	Frequency	%	Frequency	%
At most 5	64	43.5	23	19.2	87	32.6
6–10	73	49.7	35	29.2	108	40.4
11–15	8	5.4	33	27.5	41	15.4
16–20	2	1.4	22	18.3	24	9.0
21–25	-	-	6	5.0	6	2.2
Above 25	-	-	1	0.8	1	0.4
Total	147	100	120	100	267	100
Mean		7.1%		12.5%		9.5%
S.D		3.5		6.3		5.6
Skewness		0.7		0.3		0.9

**Table 4:** Variation in household food waste proportion between rural and urban households.

Rural N = 147		Urban N = 120		Statistics	
Mean	Standard deviation	Mean	Standard deviation	*Z*-value	*p*-value
0.071	0.035	0.125	0.063	2.55	0.0054 ***

*** means significant at 1%.

**Table 5:** Beta regression results.

Variable	Rural					Urban				
	Coeff.	Std. Error	z	*p*-Value	dy/dx	Coeff.	Std. Error	Z	*p*-Value	dy/dx
Sx(*X*_1_)	0.2092 ***	0.07499	2.79	0.005	0.01347	0.0611	0.09541	0.64	0.522	0.00635
AgM(*X*_2_)	0.0061	0.00497	1.23	0.218	0.00039	−0.0032	0.00604	−0.52	0.602	−0.00033
Mrst(*X*_3_)	−0.0645	0.08111	−0.79	0.427	−0.00415	0.0823	0.10593	0.78	0.437	0.00853
SchY(*X*_4_)	−0.0096	0.00940	−1.02	0.309	−0.00061	0.0082	0.01289	0.63	0.526	0.00086
WrkEx(*X*_5_)	−0.0261 ***	0.00747	−3.50	0.000	−0.00167	−0.0029	0.00768	−0.38	0.702	−0.00031
HhS(*X*_6_)	0.0199	0.02260	0.88	0.378	0.00127	−0.0155	0.02061	−0.75	0.452	−0.00162
DpdR(*X*_7_)	−0.0563 *	0.03035	−1.86	0.064	−0.00360	−0.1613 ***	0.04541	−3.55	0.000	−0.01691
MntInM(*X*_8_)	3.58 × 10^6^ **	1.43 × 10^6^	2.51	0.012	2.29 × 10^7^	1.83 × 10^6^ ***	6.49 × 10^7^	2.81	0.005	1.91 × 10^7^
HhFExpM(*X*_9_)	5.48 × 10^6^	4.27 × 10^6^	1.28	0.199	3.50 × 10^7^	5.98 × 10^6^ **	2.68 × 10^6^	2.23	0.026	6.26 × 10^7^
HhED(*X*_10_)	−0.0224	0.05565	−0.40	0.687	−0.00143	0.1744 ***	0.06449	2.70	0.007	0.01826
HhEO(*X*_11_)	−0.0265	0.03775	−0.70	0.483	−0.00169	0.1747 ***	0.04487	3.89	0.000	0.01830
TrLF(*X*_12_)	0.1765 ***	0.03640	4.85	0.000	0.01128	0.1083 ***	0.02951	3.67	0.000	0.01134
SnY(*X*_13_)	−0.00097	0.08117	−0.01	0.990	−0.00006	0.1393	0.10385	1.34	0.180	0.01439
FWFp(*X*_14_)	0.0851	0.08992	0.95	0.344	0.00534	−0.0150	0.10081	−0.15	0.882	−0.00157
_cons	−3.0166	0.27586	−10.94	0.000		−3.0352	0.37989	−7.99	0.000	
scale cons	4.3100	0.1177	36.61	0.000		3.6948	0.1295	28.53	0.000	

Rural: LR chi^2^ (14) = 60.01, Prob > chi^2^ = 0.0000, Log likelihood = 320.637. Urban: LR chi^2^ (14) = 63.71, Prob > chi^2^ = 0.0000, Log likelihood = 195.89. Note: LR means Likelihood Ratio, *, ** and *** means significant at 10%, 5% and 1%, respectively.

## Data Availability

Data are available upon request from the first author.
